# Nanostructure-directed chemical sensing: The IHSAB principle and the dynamics of acid/base-interface interaction

**DOI:** 10.3762/bjnano.4.3

**Published:** 2013-01-14

**Authors:** James L Gole, William Laminack

**Affiliations:** 1 Department of Physics, Georgia Institute of Technology, Atlanta, Georgia 30332, USA; 2 Department of Mechanical Engineering, Georgia Tech, Atlanta, Georgia 30332, USA

**Keywords:** chemical sensors, gas interface interactions, IHSAB theory, nitrated oxides, porous silicon

## Abstract

Nanostructure-decorated n*-*type semiconductor interfaces are studied in order to develop chemical sensing with nanostructured materials. We couple the tenets of acid/base chemistry with the majority charge carriers of an extrinsic semiconductor. Nanostructured islands are deposited in a process that does not require self-assembly in order to direct a dominant electron-transduction process that forms the basis for reversible chemical sensing in the absence of chemical-bond formation. Gaseous analyte interactions on a metal-oxide-decorated n-type porous silicon interface show a dynamic electron transduction to and from the interface depending upon the relative strength of the gas and metal oxides. The dynamic interaction of NO with TiO_2_, SnO_2_, NiO, Cu*_x_*O, and Au*_x_*O (*x* >> 1), in order of decreasing acidity, demonstrates this effect. Interactions with the metal-oxide-decorated interface can be modified by the in situ nitridation of the oxide nanoparticles, enhancing the basicity of the decorated interface. This process changes the interaction of the interface with the analyte. The observed change to the more basic oxinitrides does not represent a simple increase in surface basicity but appears to involve a change in molecular electronic structure, which is well explained by using the recently developed IHSAB model. The optical pumping of a TiO_2_ and TiO_2−_*_x_*N*_x_* decorated interface demonstrates a significant enhancement in the ability to sense NH_3_ and NO_2_. Comparisons to traditional metal-oxide sensors are also discussed.

## Introduction

The combination of tailored active interfaces, the ability to confine processes at the nanoscale, and the ability to manipulate nanostructured materials and their interaction at these select interfaces, offers the opportunity to develop economically viable, energy efficient, and sensitive devices for the direct sensing of a variety of chemical species. Processes that are driven by nanostructure-focused Brønsted and Lewis acid/base chemistry can provide rapidly responsive and sensitive (ppb) sensor platforms [1–4]. Within this framework, the creation of highly active, nanopore-coated microporous *extrinsic* semiconductor interfaces, their ability to provide readily accessible significant light-harvesting surface areas [5], and their ability to be transformed with selective nanostructure sites, enables sensing based on efficient electron transduction. Decorated microporous arrays enable enhanced analyte diffusion to active sites [6], whereas the nanopores provide a “phase matching” region with which the modifying nanostructured materials of interest can be made to interact in a controlled manner to promote a range of interface sensitivities.

The selection of the appropriate nanostructured materials relies primarily on the inverse hard and soft acid/base (IHSAB) model [3], based on concepts from hard and soft acid/base theory to develop model nanostructures that, within themselves or deposited on high-surface-area interfaces, (1) provide a range of selectable sensitivities [3,7] to a variety of analytes and (2) provide a sensitive and dynamic [8] mechanism for electron transduction. The IHSAB model links chemical selectivity and the mechanism of sensor response, for nanostructure-modified and -directing acidic or basic sites on microporous extrinsic semiconductor channels, through fractional deposition of the nanostructures. *These nanostructures do not form a surface coating but rather act as independent nanostructured sites capable of strongly directed interaction with a given analyte and subsequent rapid electron transduction.* In this configuration, the basic tenets of acid/base chemistry (the ability of Lewis bases to donate electrons and Lewis acids to accept electrons) and semiconductor physics can be coupled to provide a road map for the implementation of readily constructed, energy and cost efficient, rapidly responding devices that can be sensitive to the ppb level. The selection of the nanostructures that are deposited and the variable surface sensitivities that are produced, as they form in situ metal/metal-oxide deposits, can now be largely predicted. Further, the deposited sites can be modified in situ to form the corresponding oxinitrides, with a greatly increased basicity. Using a defined procedure, based on established molecular and semiconductor properties, the IHSAB model dictates the coupling of analyte/interface acid/base interactions with the properties of the majority charge carriers in an extrinsic semiconductor. When such properties are not already available, it is possible to use advanced computational chemistry approaches for their prediction, to improve our understanding of the dynamics of electron transduction across the interface, and to analyze the changes in molecular electronic structure that this process induces. In combination, this provides a focused chemistry that tailors electron flow at the interface, differentiates electron transduction versus chemisorption, and can enhance light-harvesting efficiency. This approach is now developed to the extent that the dynamics of analyte-decorating nanostructure–interface interactions and the nature of competitive electron dynamics can be evaluated [8].

We emphasize that the nanostructures are deposited to form islands on the micropores of the extrinsic semiconductor and suggest that the confined nature of these nanostructured islands is fundamental to their initial strong interaction and efficient electron transduction. The fractional deposition does not require time-consuming self-assembly within the pores of the interface, and is far simpler to implement than traditional thin film or alternative “coating” techniques. This approach creates a distinct sensor platform where the nanostructures control and focus a variable and efficient analyte interaction and the transfer of electrons to or from the extrinsic semiconductor interface. The in situ transformation of the deposited nanostructured metal oxides to their corresponding oxinitrides [9–10], as it introduces basicity, also facilitates the change of sensor response through optical pumping [11]. The extrinsic semiconductor is, however, independently variable with a distinctly different band structure and electron dynamics associated with n- or p-type doping. Although these two components are separable, they can be combined to provide an enhanced versatility versus a single or mixed metal-oxide surface coating. In concert with the IHSAB principle, this approach leads to an optimized and simpler interface. Treatment of the semiconductors with nanostructured photocatalysts can be used to facilitate the use of the system for solar pumped sensing [7,11]. The control of the interaction of targeted analytes with a specific material and the degree to which this dictates the tailoring of interfaces through the understanding of their physics and chemistry offers a uniquely defined approach to enable the selection of device materials and a general framework for the design of advanced sensor platforms.

## The sensor interface

### Micro/nanoporous semiconductor surface

**Figure 1:**
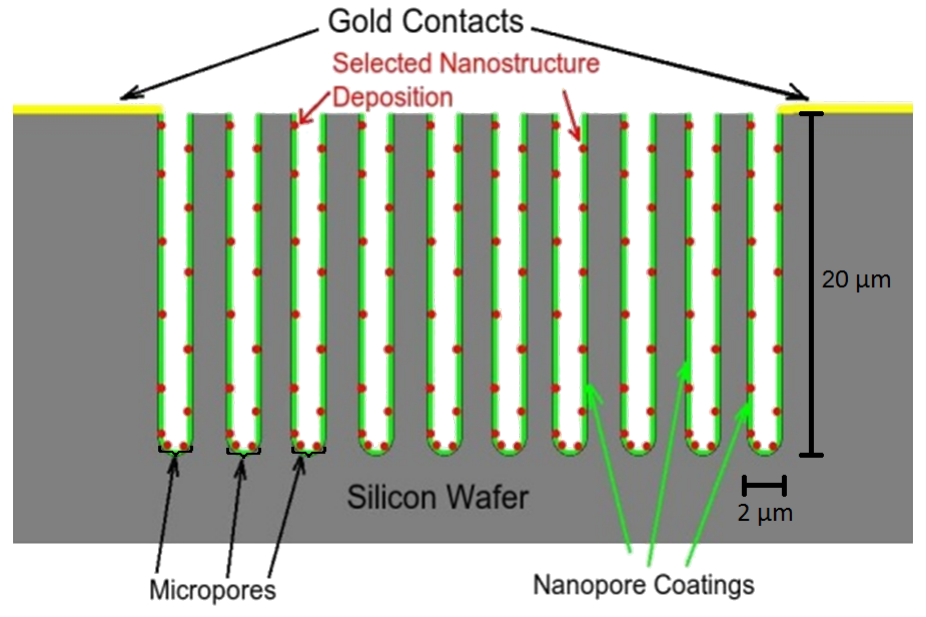
Schematic representation of PS sensor interface structure. Reproduced with permission from [3]. Copyright 2010 Wiley-VCH.

The design of the semiconductor interfaces that have been used thus far in sensor experiments is illustrated by the porous silicon (PS) nano/microporous interface depicted in Figure 1, and views of the pore structure and nanoparticle deposition are given in the experimental section. The micropores are typically of dimensions from 0.5–0.7 µm for n-type and 1–1.5 µm for p-type semiconductors. These micropores are covered by a nanoporous coating (green in Figure 1). A hybrid etch procedure is used to create this desired interfacial support structure. It is possible to replace the porous silicon (PS) structure that has been etched into a silicon wafer with any alternate extrinsic III–V (GaP or InP) or II–VI semiconductor (CdTe or ZnTe) [12] as long as the combined porous nano/microstructure can be generated in these materials.

The configuration of Figure 1 can also be reconstructed as a pass-through microporous membrane [13]. This enhances the potential range of interface combinations that can be used to form alternate extrinsic semiconductors, which can be of p*,* p^+^, or n*-*type. The nanopore covered microporous structure is created specifically to facilitate efficient gaseous diffusion (Fickian) [6] to the highly active nanostructure-modified nanoporous coating, that acts to provide a phase matching for the subsequent deposition of selected nanostructured islands (Figure 1, red dots). These surface-attached nanoparticles (see Experimental section) possess unique size-dependent and electronic-structure properties that form a basis for changing the sensitivity for exposure to specific gases relative to that of the porous silicon interface on which they are deposited. The exposure alters the conductivity of the decorated porous silicon (or an alternate extrinsic semiconductor) measured by microprobe circuitry attached to the gold contacts shown in Figure 1. The operation of this system is at room temperature, as opposed to typical metal-oxide devices. In addition, the appropriate installation of heat sinks allows operation at several hundred degrees centigrade. A developed interface, thus, can operate under conditions that are not amenable to typical metal oxide systems. When the nanoparticles are photocatalysts, the interface can also operate as a nanostructure-modified microreactor for efficient chemical transformation. When the system is operated in the electron transduction mode, the transfer of electrons to an n-type PS interface, as would occur with a basic analyte, enhances the majority carriers, which are electrons, decreases the conductometric resistance and increases conductance. The removal of electrons, as would occur with an acidic analyte, decreases the majority charge carrier concentration and the conductance and increases resistance. The opposite behavior will be observed for a p-type semiconductor interface.

### Inverse hard and soft acid/base concept

**Figure 2:**
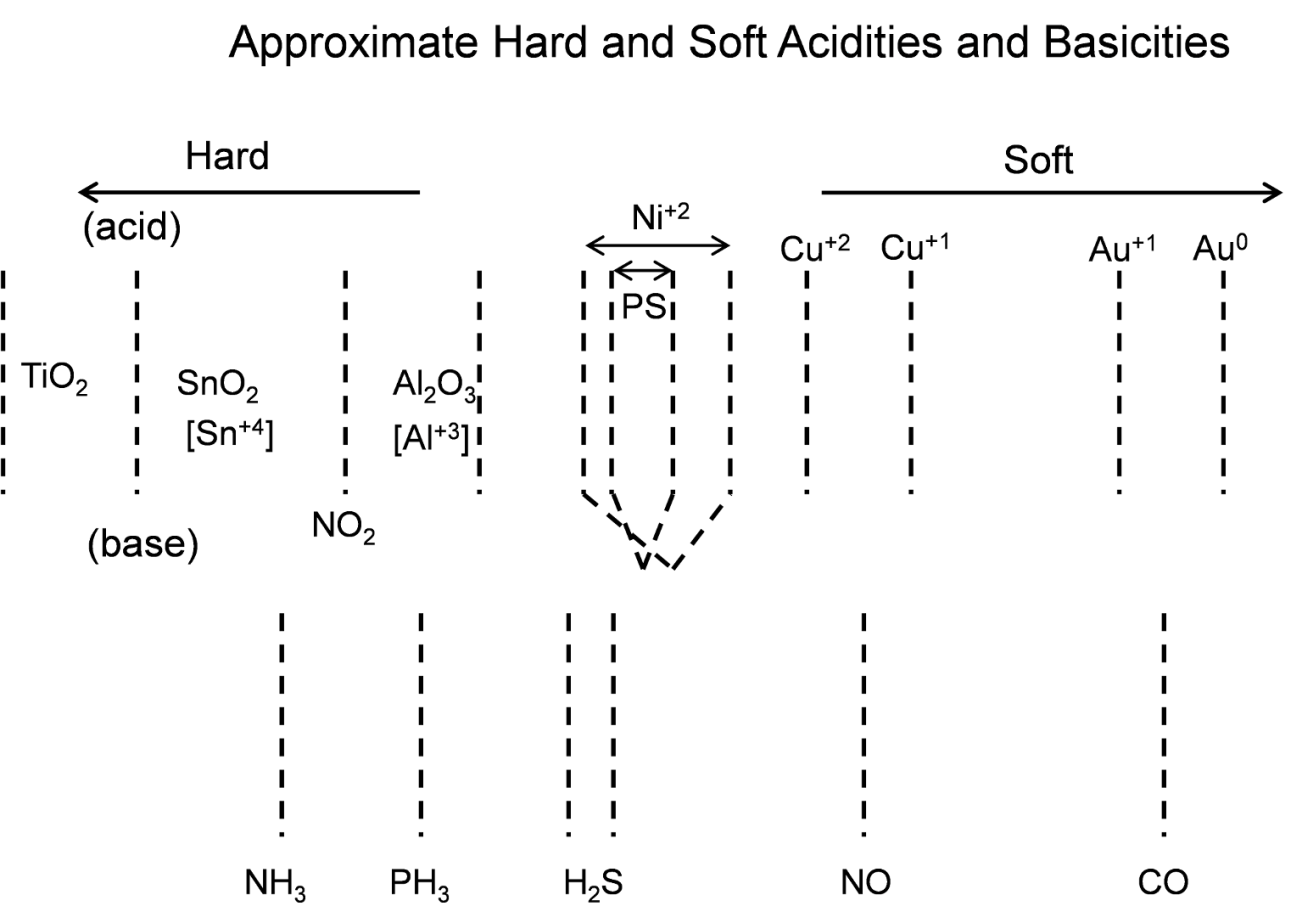
Estimated hard and soft acidities and basicities based on resistance changes relative to a p-type and n-type porous silicon interface.

We have applied the inverse hard and soft acid/base (IHSAB) concept [3,7–8], using interfaces modified by metal-oxide and nitridated-metal-oxide nanostructures. This concept, which is discussed in more detail elsewhere [3,7–8,11], complements the tenets of HSAB interactions [14–15]. It includes the coupling of analyte/interface acid/base chemistry with select interfaces, leading to a balance and separation of surface electron transduction and chemisorption, and enables the ability of active nanostructure-based sites to utilize these differences. Based on the reversible interaction of hard acids and bases with soft bases and acids, the IHSAB principle enables the selection of interacting materials that do not form strong covalent or ionic chemical bonds. It thus complements the HSAB model [14–15] for significant bond formation based on strong ionic (hard acid/base) or covalent (soft acid/base) interactions. As an extrapolation of the HSAB concept developed by Pearson [14–15] and later correlated within the context of density functional theory (DFT) by Pearson [15–18], Parr, and others [19–21], the IHSAB model is somewhat broader-based and predicts reversible sensor–analyte interactions*.* The fractional deposition of TiO_2_, SnO_2_, NiO, Cu*_x_*O, and Au*_x_*O (*x* >> 1) nanostructured islands (Figure 1) modifies the sensitivity response of the extrinsic porous silicon interface. The deposited nanostructures, in effect, dominate the PS interface on which they are deposited. The range of factors by which the response of the decorated interface to a given analyte is changed constitutes a column matrix of responses. Some typical responses, represented as the ratio of the observed signal compared to that of the untreated porous silicon interface, are summarized in Table 1 and Table 2. These ratios, while they are for a given interfacial structure [3,7], are maintained as one improves the pore structure of the interface to produce sensors that operate at the ppb level [22]. The relative reversible responses given in Table 1 and Table 2 can be correlated to allow the construction of a “Materials Positioning Diagram” for the acids and bases within the IHSAB and HSAB concepts as summarized in Figure 2 [7]. Recently, we have obtained additional data for PH_3_ on p^+^- and n-type decorated porous silicon (PS) [23]. For p-type PS, a TiO_2_ decorated surface is five times more responsive than the untreated PS interface [24].

**Table 1:** Relative increase in response (increase in resistance) of SnO_2_, NiO, Cu*_x_*O, and gold clustered oxide, Au*_x_*O treated “p-type” PS interfaces relative to the untreated PS interface. The table constitutes a response matrix to the gases PH_3_, NO, NH_3_, and SO_2_ [3,7].

	SnO_2_	NiO	Cu*_x_*O	Au*_x_*O

PH_3_	2	2.5	4	5
NO	7–10	3.5	1	1.5–2.0
NH_3_	1.5	1.5–2.0	2.0–2.5	≈3
SO_2_	4	(2)	>1	2

**Table 2:** Relative increase or decrease in resistance (decrease or increase in conductance) of TiO_2_, SnO*_x_*, NiO, Cu*_x_*O, and gold clustered oxide, Au*_x_*O treated “n-type” PS interfaces. The table constitutes a response matrix for the gases NO, NO_2_, and NH_3_. For the data presented for NO see Figure 8.

	TiO_2_	SnO_2_	NiO	Cu*_x_*O	Au*_x_*O

NO	−12^a^	−2^a^	4	1.2	1.5–2.0
NO_2_	0.75	0.5^b^	(0.9–1.0)	1	1.5–2.0^b^
NH_3_*	(3.5–4.0)	2.5	1.5	2	3

^a^indicates a decrease in resistance with analyte exposure, ^b^indicates initial response [7].

For p^+^-type PS, TiO_2_, SnO_2_, Cu*_x_*O, and Au*_x_*O decorated surfaces are respectively ≥ 4, 2.5, 3–3.5, and 7 times more responsive. The analyte response data forms the basis for the development of the materials positioning diagram [3,7] based largely on the interaction of the acidic metal oxides ranging from TiO_2_ to Au*_x_*O (*x* >> 1) and the bases NH_3_ to CO (Figure 2). The relative separation of the oxides and the bases within the range from hard to soft acids and bases dictates the observed responses of the interface. NH_3_ displays a maximum reversible response for an Au*_x_*O deposited surface whereas CO displays a maximum response for TiO_2_ and SnO_2_. Thus, in contrast to chemical bond formation, the greatest reversible response corresponds to the largest molecular orbital mismatch [3,7]. The combination of responses for the analytes considered forms the basis for selectivity based on the combinatorial arrangement of arrays of decorated n-*,* p-*,* and p^+^-type PS interfaces, for which the interfacial structure of Figure 1 can be generated.

Nitridation of the metal oxides can be used to modify the nanostructure island site basicity through in situ transformation to the corresponding oxinitrides. The degree of nitridation can be used to introduce a progressively increasing basicity. The transformation is accomplished in a manner analogous to that applied to the facile conversion of nanostructured TiO_2_ to TiO_2−_*_x_*N*_x_* [9–10]. This in situ modification shifts the positioning of the oxides toward the soft acid side of Figure 2 as it promotes the formation of a more basic interface. This enhancement of basic character promotes a significant change in sensor response.

## Results and Discussion

### Nitridation concept and enhanced basicity associated with the nitridation of oxide interfaces

The enhanced basicity inherent to the oxinitride systems that we are developing can be demonstrated in multiple ways. Consistent results are obtained from the measurement of the in situ change in response resulting from nitridation, as predicted by the IHSAB concept and its correlation with an enhanced basic character, gauged also by the softening of acidity. Further, by examining the surface chemistry of nitridated nanostructures and applying the decomposition reaction of methanol, it is possible to distinguish acid and base sites and therefore the transformation from acidic to basic sites. These studies also define a broadened interaction matrix as it extends from physisorption (sensing) applications to chemisorption and microreactor design.

**Figure 3:**
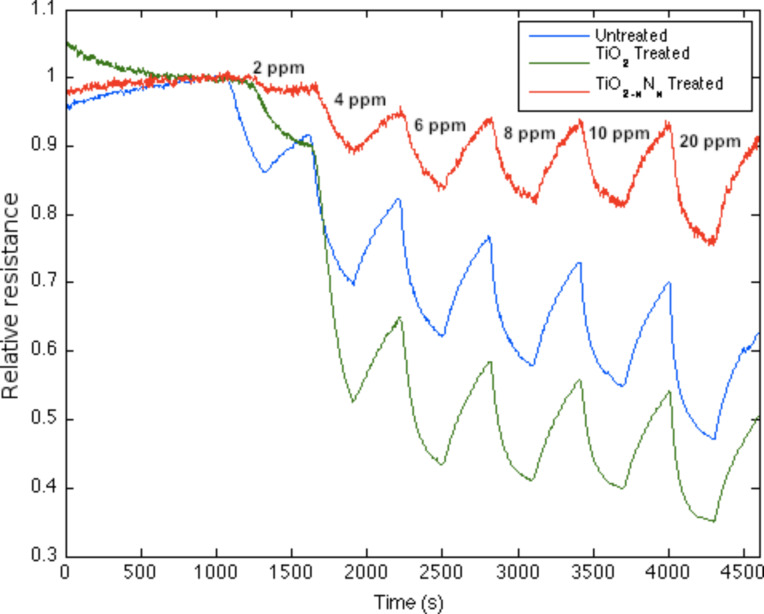
Response corresponding to decreasing resistance as NH_3_ contributes electrons to an untreated porous silicon (PS), TiO_2_, and a TiO_2−_*_x_*N*_x_* treated PS interface. The TiO_2−_*_x_*N*_x_* treated interface is basic relative to the PS and TiO_2_ treated PS acidic sites.

Recently, we have produced visible-light-absorbing TiO_2−_*_x_*N*_x_* photocatalyst nanoparticles in seconds at room temperature, using alkyl ammonium compounds [9–10,25–26], leading to the direct nitridation of highly porous TiO_2_ nanocolloids. We have found that a similar effect produces an in situ nitridation as it modifies the response of a semiconductor interface and that this effect can be explained within the IHSAB format [3,6–8,24,27–29]. Nanostructured TiO_2_ represents a strong (hard) acid. Its oxinitride, TiO_2−_*_x_*N*_x_*, once formed, through in situ treatment of a TiO_2_ deposited surface, has gained considerable basic character. The data in Figure 3 compare the response of an untreated n-type PS interface, upon exposure to 2–10 and 20 ppm of NH_3_, to that for the interface treated with a deposition of “acidic” TiO_2_ nanostructures, and this same interface where the deposited nanostructures have been converted from TiO_2_ to the more basic TiO_2−_*_x_*N*_x_*. A considerably longer time is required for the conversion of the nanostructure deposited to the PS surface.

**Figure 4:**
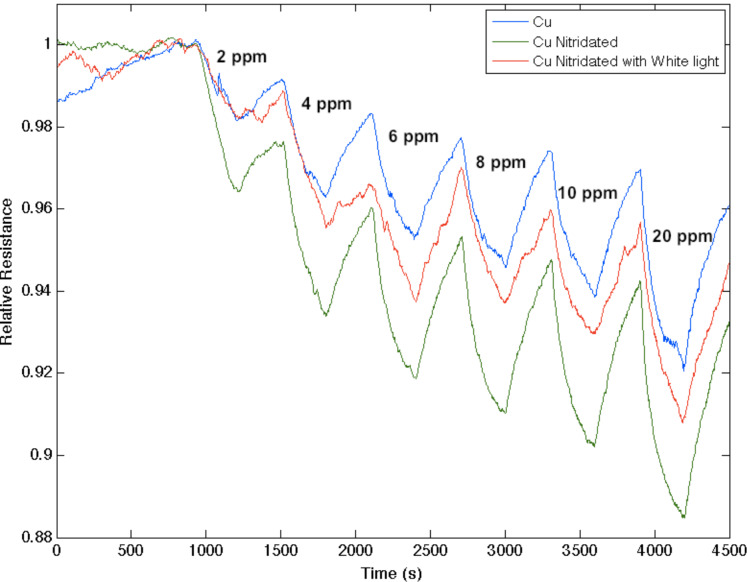
Response corresponding to decreasing resistance as NH_3_ contributes electrons to a Cu*_x_*O-treated porous silicon (blue) and nitridated Cu*_x_*O-nanostructure-treated PS (green). The nitridated Cu*_x_*O-treated interface is basic relative to the PS and Cu*_x_*O-treated PS acidic sites. This interface becomes more acidic upon exposure to white light (red).

TiO_2_, as a strong acid, enhances the capture of electrons, transferring these electrons to increase conductance (decrease resistance) relative to the undecorated interface. The more basic oxinitride does not facilitate electron transduction as efficiently and the sensor response corresponds to a conductance decrease relative to the untreated interface. Note also that the in situ nitridation of TiO_2_ shifts the nature of this metal oxide nanostructure toward the soft acid side of Figure 2, closer to ammonia. The IHSAB principle dictates [3,6–8,24,27–29] that the orbital matchup with NH_3_ is enhanced and therefore the reversible response of the TiO_2−_*_x_*N*_x_* interface should decrease relative to TiO_2_, as it does. Similar decreases in the observed sensor response are observed as nitridated SnO_2_ interacts with NH_3_ and NO with which its molecular orbital makeup now becomes more closely aligned. The nitridation of NiO also leads to a decrease in response for NO; however, the reversible response resulting from the interaction with NH_3_ increases. Figure 4 presents comparable data as 1–10 ppm of ammonia interacts with a nitridated copper-oxide-treated n-type PS interface after it has been converted in situ to a “copper oxinitride” interface. Again, the nitridation of Cu*_x_*O forms more basic sites and shifts the response of the modified nanostructures further to the soft-acid side of Figure 2. It is tempting to suggest that the formation of the oxinitride should simply increase the basicity of the nanostructure surface and thus should decrease the response to NH_3._ However, this does not occur. The nitridated copper oxide is shifted further to the soft-acid side of ammonia in Figure 2, dictating a greater molecular orbital mismatch. The IHSAB principle suggests, counter to intuition, that the response of the in situ treated nitridated copper oxide interface should increase relative to that of Cu*_x_*O, precisely as is observed. In Figure 2, NO is positioned directly under the copper oxides. Nitridation shifts the copper oxides to the soft-acid side and away from NO, leading to an increase in molecular orbital mismatch and the reversible response of the oxinitride to NO. These results strongly suggest that the IHSAB principle can be used as an important distinguishing principle of sensor response.

### Microcatalysis of metal oxide and oxinitride surfaces

We have examined several of the metal oxide and oxinitride samples for the qualitative aspects of their surface chemistry using the methanol decomposition reaction. This reaction is not a replacement for titrations with model acid and base compounds; however, the MeOH probe reaction has been effectively used by Wachs [30–31] to characterize the surface of bifunctional, mixed metal oxides. They have demonstrated the utility of evaluating redox, acid and base sites on surface-opened reaction manifolds leading to the products: formaldehyde, dimethyl ether, and CO/CO_2_, respectively.


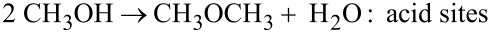



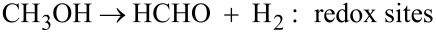



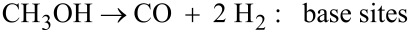


Using these probe reactions, we have discerned that the nitridation process offers the opportunity to convert the metal-oxide acid sites to metal-oxinitride surface sites.

### Optical pumping of a nanostructure-modified porous silicon interface

**Figure 5:**
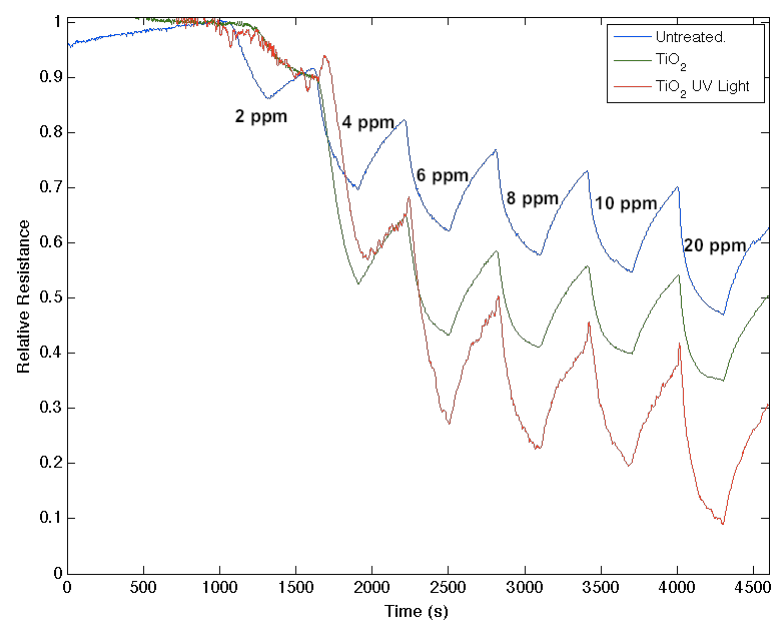
Response corresponding to decreasing resistance as NH_3_ contributes electrons to a TiO_2_ treated n-type PS interface without light exposure and the same interface exposed to UV radiation, which makes the TiO_2_ more acidic, increasing electron extraction and conductance.

It is possible to enhance the sensitivity of an n-type extrinsic semiconductor PS interface to which TiO_2_ and TiO_2−_*_x_*N*_x_* photocatalytic nanostructures have been deposited. PS sensor interfaces can be treated to form TiO_2_ nanostructure island sites that greatly enhance the surface acidity and sensitivity to NH_3_. Figure 5 represents the response of an n-type sensor to NH_3_ and demonstrates that the sensitivity to NH_3_ greatly increases as UV light impingent on the sensor increases the acidic character of TiO_2_. Here, ammonia contributes electrons to the PS interface resulting in an increase in the majority carriers and therefore an increase in conductance. This is manifest by a decreasing resistance. While the response to NH_3_ is rapid [1,3,8,11] in this unsaturated mode, the system recovery is slowed as NH_3_, a sticky gas, results in a drift in baseline (see Experimental section).

**Figure 6:**
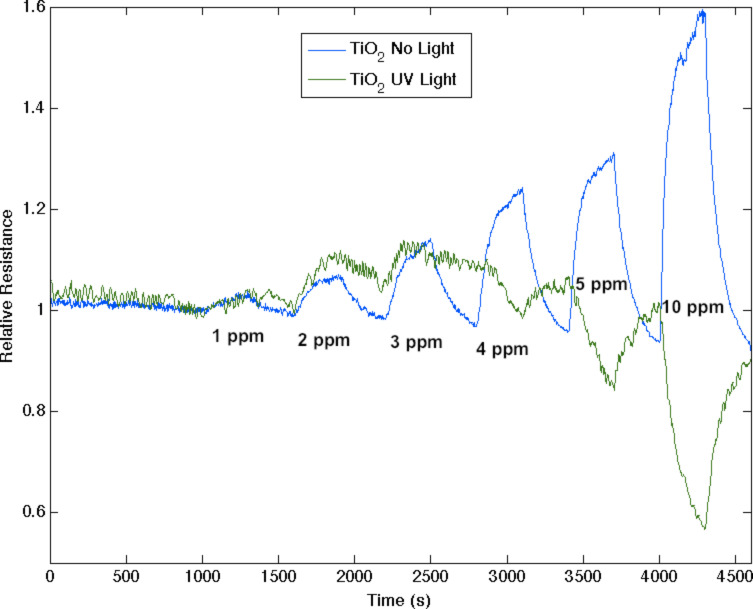
Response corresponding to increased resistance as NO_2_ extracts electrons from a TiO_2_ treated n-type PS interface without light exposure (blue) and the same interface exposed to UV radiation (green). Note that the response reverses with light exposure indicating that a more acidic TiO_2_ is now extracting electrons.

**Figure 7:**
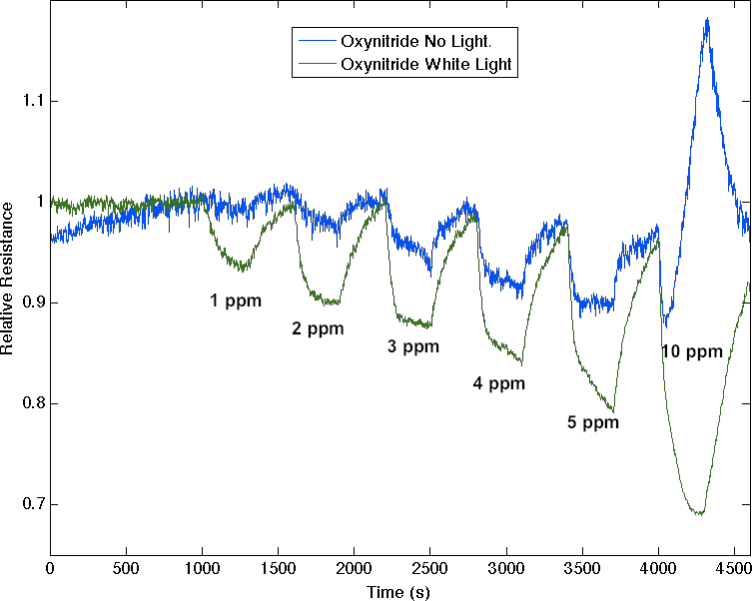
Response corresponding to decreasing resistance as a significant TiO_2−_*_x_*N*_x_* fractional nanostructure deposition leads to the extraction of electrons from the moderate acid NO_2_ for all but the highest NO_2_ concentrations (blue). The efficiency of electron extraction is less than that of TiO_2_ at a similar concentration. Response corresponding to a substantially greater decrease in resistance upon white-light exposure (green).

NO_2_, as a moderate acid, is found to extract electrons from a PS interface [7,29] increasing majority carrier “holes”, and treatment with moderate concentrations of TiO_2_ enhances the response to NO_2_. However, Figure 6 demonstrates that UV light now reverses this process as the optically pumped TiO_2_ treated interface becomes more acidic and begins to extract electrons from the moderately acidic NO_2_. In situ nitridation of the TiO_2_ to form the oxinitride, TiO_2−_*_x_*N*_x_* [9–11], enhances the visible-light response, basicity and sensitivity of the decorated PS interface. At low fractional TiO_2_ depositions, NO_2_ dominates TiO_2−_*_x_*N*_x_* [11] and white-light excitation increases the sensor response in the form of an increased resistance. In contrast, at higher fractional TiO_2−_*_x_*N*_x_* depositions, Figure 7 demonstrates that white light now increases the sensor response in the form of an increased conductance as the TiO_2−_*_x_*N*_x_* [11] decorated interface is found to extract electrons. (Note that the baseline drift in Figure 6 and Figure 7 is small). With light intensities *less than a few lumens per (centimeter squared sterad nanometer)*, responses are enhanced by up to 150% through interaction with visible (and UV) radiation. These light intensities should be compared to the sun’s average spectral brightness, ≈500 lm/(cm^2^·sr·nm), suggesting an important extension of the IHSAB principle and the possibility of *solar pumped sensing.* In these experiments, one must be concerned with the photocorrosion of silicon and the presence of water in the pores at room temperature. For this reason, the sensors are thoroughly washed in methanol, dried and kept in a desiccator (see Experimental section) between uses. After exposure to the light levels in these experiments, the sensors are found to respond as they did before light exposure. The results we obtain with optical pumping not only follow the tenets of the IHSAB principle but they suggest an intriguing electron dynamics that pervades through these systems.

### Dynamics of nanostructured metal oxide analyte interaction

We have previously considered the dynamic interaction and competition between NO_2_ and a TiO_2_-nanostructure-modified n*-*type PS interface. The addition of NO_2_, which extracts electrons, leads to a resistance increase (conductance decrease). However, a fractionally deposited strong acid such as TiO_2_ can compete effectively with the moderately strong acid, NO_2_, for the available electrons in this system [3,8]. As NO_2_ is introduced to the decorated PS interface and attempts to extract electrons, the sensor resistance rises rapidly to a point where the electron depletion reaches a limiting value as nanostructured TiO_2_ islands coupled to the PS interface prevent further electron withdrawal and reverse the flow of electrons so as to increase the donor and conduction-level electron concentrations. This can lead to a sharp decrease in the resistance. The process of interaction is a dynamic one as TiO_2_ and NO_2_ vie for the available electrons as the NO_2_ is introduced and removed from the system. Furthermore, the process of electron withdrawal is strongly influenced by the relative concentration of the TiO_2_ island sites. A similar dynamic playoff is observed with the amphoteric NO radical, which can be either a weak acid or a weak base [7,29].

**Figure 8:**
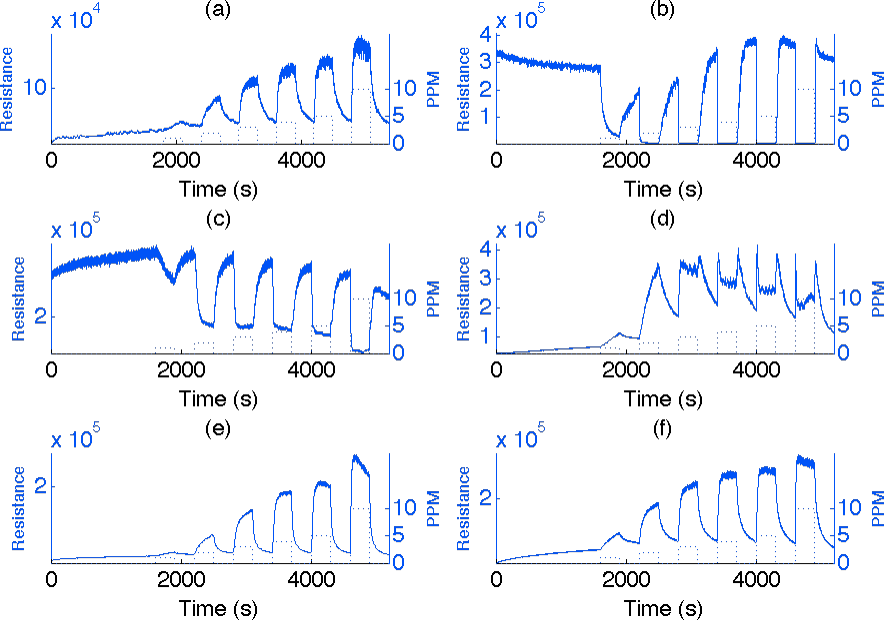
Comparison of responses to 1–5 and 10 ppm NO for (a) an untreated n-type PS micro/nanostructured interface with those treated with (b) TiO_2,_ (c) SnO_2_, (d) NiO, (e) Cu*_x_*O, and Au*_x_*O fractional nanostructured island depositions. NO was pulsed onto these interfaces with a 300 s half-cycle followed by a 300 s half-cycle nitrogen cleaning. The system was purged with UHP nitrogen for 1800 s before operation.

Figure 8a shows a positive resistance change with concentration, indicating that NO acts like a weak acid when brought in contact with an n*-*type PS interface. The boxes in the figure correspond to the concentration range from 1 to 5 and 10 ppm. Nanostructure-treated PS interfaces deposited with the acidic metal oxides in decreasing strength TiO_2_ > SnO*_x_* > NiO > Cu*_x_*O > Au*_x_*O (*x* >> 1) demonstrate not only a starkly different response but also clear trends that can be associated with the relative acid strengths of the metal oxides. Figure 8b and Figure 8c indicate the responses for the PS interfaces decorated with TiO_2_ and SnO*_x_*. The strong acid character of TiO_2_ and to a lesser degree SnO_2_ has overcome the ability of NO to extract electrons. Instead, electrons are extracted from NO and transferred to the PS interface to greatly increase conductance. Although the observed responses are virtually linear to 5 ppm for the untreated PS interface, they are clearly quenched for the TiO_2_ and SnO_2_ surfaces at NO concentrations in excess of 4 ppm. The observed response at 10 ppm diminishes for TiO_2_, which suggests that the ability of this interface to extract electrons from NO has reached a limiting value. This is less apparent for SnO_2_, but the ability of the interface to extract electrons is still significantly diminished. The magnitude of the signals observed for the TiO_2_ and SnO_2_ interfaces, while opposite in sense, exceed those for the untreated PS interface by factors of 12 and 2, respectively [7,29].

Figure 8d corresponds to the response of a PS interface treated with the intermediate acid NiO. The response displays an intriguing intermediate behavior, as NO and the decorated PS interface now compete effectively for electrons. At the lowest NO concentrations the ratio of the responses (2 ppm/1 ppm) after NiO treatment is enhanced significantly relative to the response of the untreated n-type PS interface and the observed response rises to a maximum throughout the cycle of NO exposure. For NO concentrations in excess of 3 ppm, the dynamic response is found to rapidly increase to a sharp maximum, subsequently decrease, oscillate and then increase as the NO concentration decreases. At 4, 5, and 10 ppm the dynamic behavior is even more pronounced. The transfer of electrons to NO increases to a maximum, indicated by the spike-like features, which diminish in width with increasing NO concentration. We have suggested that the transfer of electrons to NO reaches a limit when the n-type PS interface is sufficiently depleted such that it acts as a stronger acid than the NO radical [7]. Electrons are extracted from NO (acting as a base), and the dynamic resistance (increase in conductance) decreases as the semiconductor donor levels are repopulated. The oscillatory behavior, which is especially apparent at concentrations of 4, 5, and 10 ppm, is suggested to result from a continual if not less pronounced change of the competing NiO-decorated interface and NO. The time-dependent competition between NO and the NiO-decorated interface means that the sensors return slowly to a baseline response. This will be the subject of further study.

Figure 8e and Figure 8f demonstrate a clear trend in the responses of the weaker acid nanostructure deposited PS interfaces, based on Cu*_x_*O and Au*_x_*O nanostructure depositions and an increase in the response of the PS interface by factors of 1.2 and 1.5, respectively [7,29]. The increase in response is, as expected, greater for the weaker acid Au*_x_*O. The Cu*_x_*O and Au*_x_*O decorated interfaces thus act to enhance the electron-withdrawing power of the NO radical, which suggests that they represent weaker acids on the n-type PS interface. Consistent with trends in the acid strength of the nanostructure deposits, the Cu*_x_*O response increases to a maximum with NO exposure for concentrations 1–4 ppm; however, this increase is slowed at 4 ppm. At 5 ppm the response peaks at an intermediate time for the NO exposure, and at 10 ppm the signal peaks shortly after the NO exposure and, counter to the behavior at lower pressures, decreases. This overall behavior is consistent with the weaker acid nature of Cu*_x_*O versus NiO. At 4 ppm, the Cu*_x_*O-decorated interface begins to compete effectively with NO for the available electrons, and at 10 ppm the interface is able to overcome the extraction of electrons by NO as the response shows a decrease with time of NO exposure. Note also the almost complete return to baseline.

The trends that we describe for Cu*_x_*O extend to the weaker acid Au*_x_*O-decorated interface. Here, Figure 8f demonstrates that Au*_x_*O response increases to a maximum with exposure to NO concentrations 1–4 ppm. At 5 ppm the response peaks at an intermediate response to the NO exposure. However, at 10 ppm the response decreases with NO exposure but at a much slower rate than does the response for Cu*_x_*O. The extraction of electrons by NO is overcome but to a much lesser extent. Note also the return to baseline.

We suggest that the important role played by the deposited nanostructures and the nature of the acid/base interaction that they direct play a significant role as the variations in response of the nanostructure-treated PS interfaces reflect changes in the donor level population. These trends are also observed for the relative responses as NO interacts with p-type PS. Within this framework the behavior of the NiO-decorated interface represents a distinct intermediate behavior. The NiO nanostructure-deposited interface responds similarly to that of the weak acids Cu*_x_*O and Au*_x_*O at low analyte concentrations; however, the NiO-treated interface begins to approach a surface similar to that treated with the stronger nanostructured acids TiO_2_ and SnO*_x_* at higher concentrations. When interacting with the NiO-treated interface, NO can vary from an acid to a base as a function of concentration.

### Comparison to traditional metal-oxide sensor systems

**Figure 9:**
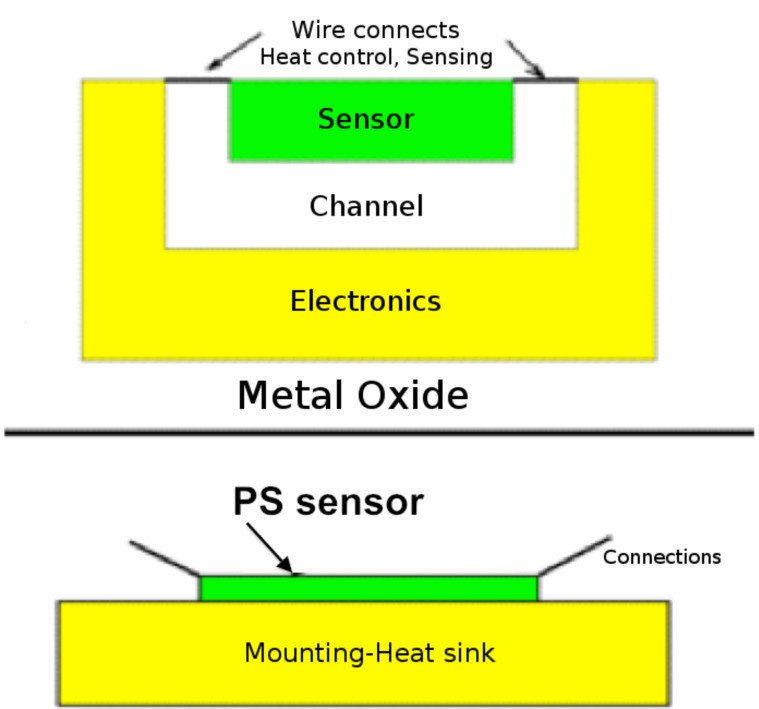
Comparison of a metal-oxide (usually SnO_2_ or WO_3_) elevated-temperature (150–500 °C) heat-controlled sensor separated from its electronics by a channel, with a heat- sunk PS sensor operating at room temperature and capable of operation to temperatures of at least 80 °C.

In contrast to traditional metal-oxide devices, the present systems create a dual interface where the nanostructured islands and the extrinsic semiconductor act separately but are coupled. The design, operative at room temperature, adds considerable flexibility not possible in a singly or multiply “coated” metal-oxide interface. Metal-oxide sensors (Figure 9) (when compared also to electrochemical sensors) are slightly less costly to produce; however, concerns may include poor sensitivity, high power requirements, and most importantly, the need to operate the sensor element at elevated and controlled temperatures. A power-consuming heating element must be provided with the sensor housing to precisely control the temperature of the sensor element. This temperature control is correlated to the correct identification of the gas of interest. To distinguish one gas from another, the heating element and sensor should be well separated (channel) from the remaining electronics. In this configuration, the sensor element can be greatly affected by an impinging combustion or flue gas, rendering difficult the correct identification of gaseous species in the flow. In contrast, the PS sensor configuration depicted in Figure 1 consumes less power, is far simpler, and does not require the complexity of a system-separated sensor/heater configuration. In a heat-sunk environment (Figure 9), it is potentially capable of operation in a high-temperature gas flow.

### Future considerations development of material selection tables

It would seem appropriate to expand the selective deposition of nanostructured materials to create inexpensive microfabricated sensor platforms and develop *“material selection tables”* built on the IHSAB model. The response data that we have outlined form the basis for the development of an initial materials-positioning diagram (Figure 2) predicted by the IHSAB concept. It remains to expand the metal-oxide data base, including the in situ transformation to the corresponding oxinitrides, and to enhance the array of distinct responses that can be developed and extended to form *“materials sensitivity matrices”* for a given analyte. This will enhance the capability to sense analytes and their mixtures. This ready transformation is easily accomplished in a manner analogous to that applied to the facile conversion of TiO_2_ to TiO_2−_*_x_*N*_x_* [9–10]. Because the in situ formation of the oxinitrides will shift the positioning of the oxides toward the soft acid side of Figure 2, it will add a notable flexibility to the materials sensitivity table. Initial results suggest that the nitridation process does not simply increase the basic character of the nanostructure surfaces but that it modifies the molecular structure and interaction as the metal-oxide deposited surface has gained considerable basic character. Initial results suggest that the nitridation process does not simply increase the basic character of the nanostructure surfaces but that it modifies the molecular structure and interaction, consistent with the IHSAB principle. This means that the sensitivity of the weaker metal oxides is enhanced by nitridation. Further, this process can be applied to create several potential visible-light-absorbing photocatalysts similar to TiO_2−_*_x_*N*_x_* [9–10].

We wish to better understand the change in electronic character of the sensing system when it interacts with an analyte and the analyte injects or removes charge from the semiconductor interface to change the resistance. It is of interest to understand how the occupied bands in the semiconductor change when the analyte interacts with the surface. How does the change in the bands occur? How does this affect the band gap? The prediction of the electronic properties, especially the band gaps, is closely tied to the actual structure of the interface. Where does the analyte bind to the interface? What types of interactions dominate the analyte–interface bonding in, for example, the competition between physisorption (electron transduction) and chemisorption? The IHSAB concept appears to map a general approach to the development of sensor systems; however, it remains to obtain a more quantitative picture of these systems.

## Conclusion

We have demonstrated the efficacy of fractional nanostructure depositions as a means of obtaining distinct sensor responses which show the potential for combination in an array-based format. The behavior of these systems appears to be well represented by the newly developing IHSAB model. We have also considered the conversion of the metal oxides in situ to their oxinitrides and the enhanced basicity that this introduces to a nanostructure-decorated PS interface. These systems also display time-dependent dynamics, which must be incorporated into the IHSAB model. This will be the subject of future studies.

## Experimental

**Figure 10:**
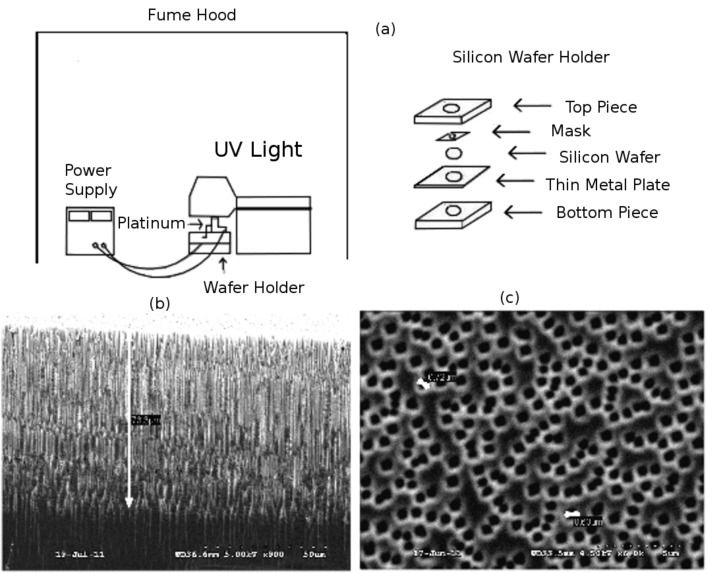
(a) Schematic view of apparatus used to generate the micro/nanoporous structure in n-type silicon. (b) top and (c) side view of the pores. Reproduced with permission from [7]. Copyright 2012 Wiley-VCH.

**Figure 11:**
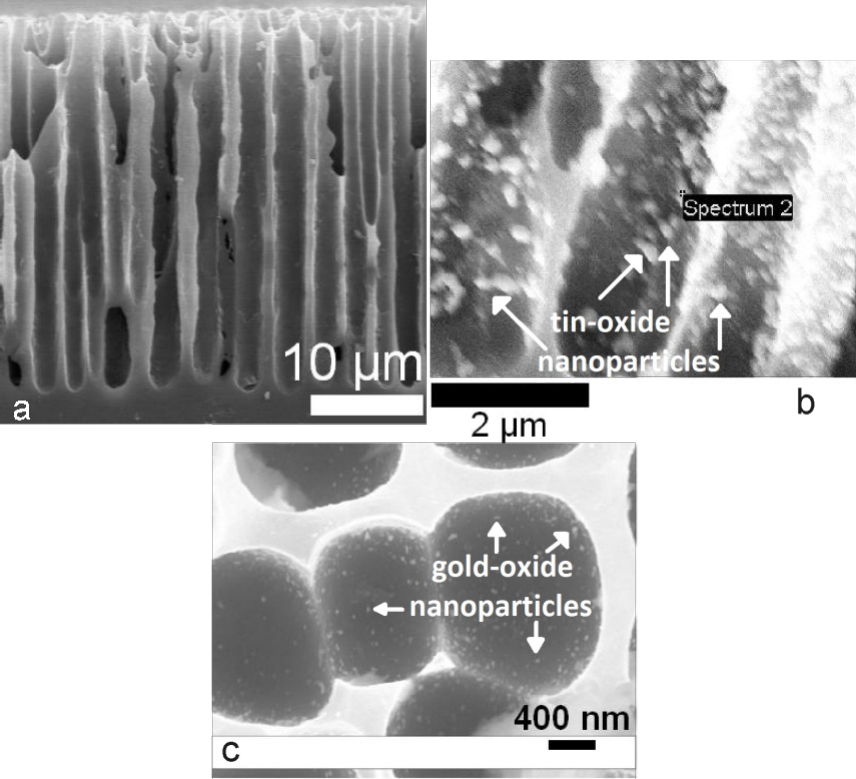
(a) Close up side view of hybrid porous silicon film. (b) 10–100 nm SnO*_x_* nanoparticle tin-oxide deposits on porous silicon micropores; (c) 10 to 30 nm Au*_x_*O nanostructures on porous silicon. Reproduced with permission from [3]. Copyright 2010 Wiley-VCH.

As described previously [7], highly efficient nanostructure-modified interfaces on either p- or n-type PS are produced, as we generate the micro/nanoporous interface outlined in Figure 1 [3]. A hybrid etch procedure is used to generate nanopore-covered micropores exemplified in Figure 10 and Figure 11 [3,7,13]. The PS interface is generated by electrochemical anodization of 1–20 Ω·cm, n-type (phosphorous-doped) silicon(100) wafers (Wafer World) or 7–13 Ω·cm (Figure 10), p-type (boron-doped) silicon(100) wafers (Siltronix) (Figure 11). The anodization of the n-type wafers [31–32] is done under topside illumination by using a Blak-Ray mercury lamp. The silicon wafer is etched in a 1:1 solution of HF and ethanol at a current between 8–15 mA/cm [27–28,32–33]. The anodized n-type sample is placed in methanol for a short period and subsequently transferred to a dilute HF solution for a 30 minute period and then washed again in methanol. This process creates a porous structure with pore diameters of order 0.5–0.7µm and pore depths varying from 50 to 75 µm (Figure 10). The p-type wafers are etched in 1 M HF, and 0.1 M tetrabutylammonium perchlorate (TBAP) in acetonitrile (MeCN) at 3–6 mA/cm^2^. The anodized sample is cleaned in MeCN for 10 minutes to purge any residue in the pores due to the etch solution [3,27,34]. Subsequently, it is immersed for several minutes in HF and then methanol. The PS has a porosity of 50–80% with the micropore diameters varying from 0.8 to 1.5 µm and pore depths varying from 10 to 30 µm. The micropores provide a medium for Fickian diffusion to the surface nanoporous layer.

Before the anodizations, an insulation layer of SiC (≈1000 angstroms) is coated onto the c-Si substrate by PEVCD methods. Windows of size 2 × 5 mm are opened in this layer by Reactive Ion Etching (RIE). The SiC layer serves two purposes: SiC makes it possible to form the hybrid micro/nanoporous PS structure in the 2 × 5 mm windows during electrochemical anodization because of its resistance to HF. The SiC also aids the placement of gold contacts exclusively on the porous layer for resistance measurements and acts as an electrical insulator on the doped silicon. The PS hybrid arrays of nanopore-covered micropores are tested at room temperature for their individual sensor response. The nature of this response is based on the application of the IHSAB acid/base principle. The selection of the nanostructures and the variable surface sensitivities that are produced as they form in situ metal-oxide deposits introduces a distinct systematics of design, which can be predictably formatted. The approach is unique in that the nanostructures are deposited fractionally to the PS micropores and this fractional deposition DOES NOT require any time-consuming self-assembly within the pores. This is not a coating technique or one that requires an exacting structural film arrangement but is, in fact, a much simpler process. The requirement is that the nanostructure deposition must be maintained at a sufficiently low level to avoid cross-talk between the nanostructures (Figure 11) that, as it increases, leads to a noisy device and the eventual loss of functionality. The time for exposure of the pore structure to the nanostructure-forming solutions is sufficiently short (10–30 sec.) that the depositions represent an upper bound. If the deposition exceeds the concentration where the nanostructures begin to interact, the observed conductometric signals will display instability. Together, the combination of the distinctly different responses observed can be used as a basis to develop selectivity. Results obtained with nanostructured deposits generated from electroless gold, tin, nickel and copper, as well as nanotitania are considered in this study.

With the exception of the gold depositions, all of the nanostructured metals deposited to the PS surface are readily oxidized to SnO*_x_* (*x* = 2,4) and Cu*_x_*O (*x* = 1,2) as demonstrated by XPS measurements [23]. The initially introduced titania (anatase) may be crystalline; however, we cannot be certain of this crystallinity after deposition to the PS interface. The untreated PS hybrid structures are exposed to the electroless solutions for 10 to 30 seconds and are placed in DI H_2_O and MeOH for consecutive 120 second periods. The oxidized electroless metal depositions when characterized before deposition correspond to amorphous structures displaying no diffraction patterns. Therefore, it is difficult to envision their crystallization during the short deposition and subsequent surface cleaning process. After deposition, the decorated surfaces are cleaned for 120 s in DI and 120 s in methanol. Basic character is introduced to the nanostructured metal oxides by direct in situ treatment with triethylamine (TEA). The metal-oxide treated surface is exposed to the TEA for 10 s. The treated interface is subsequently washed in methanol to remove excess TEA and allowed to age for approximately 24 h.

The sensors are evaluated in an unsaturated mode since the time scale for reversibility may become an issue in a long-term saturated mode and the longer term exposures are not necessary. The sensor response and recovery times for “sticky gases” such as ammonia are distinctly different and full time recovery from the gas exposure takes longer than 300 s, i.e., the exposure time duration in the present configuration (Figure 2 in [1]). However, the onset of the sensor response for these atmospheric-pressure open-inlet studies remains clearly visible. This behavior, which looks very like the reverse of Figure 2 in [1] suggests that the responses for NH_3_ on PS (Figure 5) are that of a gas whose interaction may be dominated by physisorption but which also displays weak chemisorption. Purging the sensor surface with UHP N_2_ for longer durations improves the gradual shift to the initial base line. The return to baseline can also be further improved by more tightly constraining the gas flow path to the sensor surface.

In all cases, the analyte gas being sensed is brought to the hybrid surface after entrainment at room temperature in UHP nitrogen (Matheson 99.999+ %). The system is purged with UHP nitrogen for a minimum of 30 minutes before use. The typical resistances for the base PS structures range between 300 and 10,000 Ω at room temperature. The gas flow for the analyte and the entraining UHP nitrogen is controlled by MKS type 1179A mass-flow controllers. The mass-flow controllers used to control the analyte gas and the entraining nitrogen flow responded in less than 2 seconds. The diffusion time of the analyte gas to the sensors, which provides the longest system time constant, varies from four to five seconds for the lowest analyte concentrations to on the order of 1 to 2 seconds for concentrations greater than 2 ppm. These are the delay times for the observation of a signal due to the analyte in the supply line. The sensors respond to the analyte gas on a time scale of much less than two seconds. The change in resistance is measured in one-second intervals by using a DC current. The voltage bias used in these experiments is 3 V to obtain an optimum signal-to-noise ratio. A NI DAQPad-6015 is used for gathering data and supplying the DC current. Labview software is used to control the experiment and record the results. MATLAB is used in the analysis of the data.
